# Using Renal Elastography to Predict the Therapeutic Response of Nephrological Patients

**DOI:** 10.3390/jcm12237385

**Published:** 2023-11-29

**Authors:** Nicoletta Mancianti, Guido Garosi, Ernesto Iadanza, Sergio Antonio Tripodi, Andrea Guarnieri, Massimo Belluardo, Edoardo La Porta, Marta Calatroni, Maria Antonietta Mazzei, Palmino Sacco

**Affiliations:** 1 Department of Medical Science, Nephrology, Dialysis and Transplantation Unit, University Hospital of Siena, 53100 Siena, Italy; g.garosi@ao-siena.toscana.it (G.G.); andrea.guarnieri@ao-siena.toscana.it (A.G.); massimo.belluardo@ao-siena.toscana.it (M.B.); 2 Department of Medical Biotechnologies, University of Siena, 53100 Siena, Italy; ernesto.iadanza@unisi.it; 3 Department of Oncology, Pathology Unit, University Hospital of Siena, 53100 Siena, Italy; tripodis@unisi.it; 4 UO Nephrology Dialysis and Transplant, IRCCS Istituto Giannina Gaslini, 16147 Genoa, Italy; edoardo01laporta@gmail.com; 5 Nephrology and Dialysis Division, IRCCS Humanitas Research Hospital, Rozzano, 20089 Milan, Italy; marta.calatroni@hunimed.eu; 6 Department of Medical, Surgical and Neurosciences and of Radiological Sciences, Unit of Diagnostic Imaging, University Hospital of Siena, 53100 Siena, Italy; mariaantonietta.mazzei@unisi.it (M.A.M.); p.sacco@ao-siena.toscana.it (P.S.)

**Keywords:** renal elastography, chronic renal histological damage, kidney biopsy, Sethi score, shear wave elastography, renal prognosis, glomerulonephritis, acute kidney injury, acute kidney disease

## Abstract

Background: The standard method for assessing chronic renal damage is renal biopsy, which has limitations due to its invasiveness. Ultrasound elastography is a non-invasive technique that quantifies tissue elasticity and can be used to determine Young’s modulus (YM). Although this breakthrough technology has been successfully employed to evaluate liver stiffness and the extent of fibrosis, its application in kidney-related conditions still needs improvement. Methods: Our study aimed to verify the correlation between renal elastography and the chronic histological score determined via renal biopsy, evaluate the correlation between elastography and response to treatment in the short-term follow-up (6 months), and compare elastography data between renal disease patients (AKD-P) and healthy controls (HP). Results: The analyzed population consisted of 82 patients (41 HP and 41 AKD-P). The AKD-P were divided into responders (R) or non-responders (NR) based on the criteria established by the guidelines. No association was found between renal stiffness and chronic histological score. Elastography data revealed median YM values of 6.15 kPa for AKD-P and 12.2 kPa for HP, with a statistically significant difference. The median YM values of the R and NR groups were 7.4 KPa and 5.6 KPa, respectively (*p* = 0.037). Conclusions: Patient responsiveness was associated with YM, with lower values observed in the NR group. We also found that the healthy controls exhibited significantly higher YM values than the renal disease population.

## 1. Introduction

Treatment efficacy for kidney-related conditions is contingent upon the specific histology pattern revealed by a patient’s biopsy. The current standard method for diagnosing chronic renal histological damage is renal biopsy [1]. The Mayo Clinic has created the Sethi score, which is a system used to grade chronic kidney disease in the renal compartments, including glomerulosclerosis, tubular atrophy, interstitial fibrosis, and arteriosclerosis (Table 1) [2]. Although widely used in routine kidney evaluations, renal biopsies have limitations. Firstly, a renal biopsy is an invasive procedure that can result in complications such as bleeding and pain. Performing multiple kidney biopsies presents challenges. Additionally, fibrosis may develop in a patchy manner due to focal injuries, making it difficult to assess an overall fibrosis score accurately. Lastly, the acute inflammatory components are challenging to quantify histologically [3]. Moreover, physicians require a sample that effectively showcases the renal cortex, characterized by at least 10 glomeruli and two arteries [4]. The feasibility of this procedure is another element to consider. An advanced medical center with specialized equipment, skilled medical professionals, and a histopathology laboratory is required. Exploring rapid, repeatable, and non-invasive methods for evaluating the chronic component, mainly when limited laboratory resources are available, is also essential. With the advent of ultrasound elastography, we now have a new way in which to measure tissue elasticity. This innovative process allows one to calculate the physical measure of tissue stiffness, known as Young’s modulus (YM). By subjecting the tissue to stress and measuring the resulting effort, we can determine the modulus by dividing the applied stress by the induced strain expressed in pressure units. This breakthrough in medical technology opens new possibilities for understanding and treating various health conditions. Different elastography techniques can be used depending on the external force applied. The most reliable dynamic method, Shear Wave Elastography (SWE), uses ultrasound to represent the tissue’s stress under examination. The shear wave speed depends on the tissue’s elastic and viscoelastic properties and allows for the numerical estimation of YM [5,6]. Elastography (Fibroscan) is a non-invasive method currently used in clinical practice, together with serum markers, to evaluate liver stiffness and the degree of fibrosis, with a class Ia recommendation in the guidelines of the EASL (European Association for the Study of the Liver). In the hepatological field, elastography helps differentiate degrees of fibrosis according to a histological score (METAVIR score), which distinguishes five degrees, ranging from F0 (absence of fibrosis) to F4 (cirrhosis) [7]. Although there have been some initial studies, the use of elastography to assess the inflammatory and fibrotic condition of the kidneys is still in its early stages [8]. Furthermore, there is no specific recommendation regarding its application, and the available data may be limited.

### Objectives of the Study

Our study has three main goals:

1. Verify the correlation between renal elastography and the chronic histological score (Sethi score, Table 1) determined from a renal biopsy;
2. Evaluate the correlation between elastography and response to treatment in the short-term follow-up (6 months);
3. Compare the elastography data of our population with renal disease with those obtained from a healthy control group.

## 2. Materials and Method

This study was conducted prospectively. The study included 50 acute kidney disease patients (AKD-P) and a control group of 50 healthy volunteer patients (HP). The AKD-P cohort comprised patients from the Nephrology Unit who required renal biopsy due to kidney damage and met the eligibility criteria for the study. This selection procedure was carried out between May 2021 and February 2023. The HP group, on the other hand, consisted of healthy volunteers from the radiology unit who were recruited during the same study period.

Nine AKD-P were excluded from the analysis because the biopsy sample consisted of fewer than 10 glomeruli (the minimum sample required for analyzing the Sethi chronicity score). The AKD-P underwent a renal biopsy on the same day as the elastography and chemistry tests to compare the data. Nine HP were excluded from B-mode ultrasound evaluation; the presence of cysts, stones, and renal masses was accounted for, as their presence would have affected the accuracy of the elastography. The population analyzed comprised, therefore, 82 patients (41 AKD-P and 41 HP).

Three main medical figures were involved in the study: the nephrologist who performed the renal biopsy, the histopathologist (>10 years of experience in the nephrology field) who performed the analysis of the biopsy sample, and the radiologist (>5 years of experience in elastography) who performed the elastography examination.

To ensure the accuracy of the results, both the pathologist and the radiologist worked blindly, without knowing the respective results, to avoid any potential influences. This strategy was implemented to eliminate any bias and ensure that the results were solely based on the examination findings. To ensure the consistency of the methodology, a control population of HP underwent renal elastography using the same equipment as that used for the patients with kidney disease. This helped to standardize the procedure and ensure that any differences in the results were solely due to the presence of kidney disease in the patients.

The inclusion criteria were as follows:

- Age over 18 years;
- Indications for a renal biopsy (only for AKD-P) represented by one or more of the following elements: hematuria of presumed renal origin (generally in association with other significant elements such as proteinuria, hypertension, and serum biomarkers such as ANCA), significant proteinuria (>1 g/day), renal involvement in systemic disease, and renal impairment of unexplained origin.

The exclusion criteria were as follows:

- Contraindications for performing a renal needle biopsy (only for AKD-P) (renal morphological anomalies, coagulation disorders, hypertensive status that cannot be controlled pharmacologically, and non-compliant patients)
- Lack of informed consent for carrying out the tests;
- Congenital anomaly or hereditary kidney disease, vesicoureteral reflux, or hydronephrosis.

The study variables for the AKD-P were as follows:

1. Demographic-anamnestic-clinic data: age, gender, and renal disease.
2. Laboratory data: serum creatinine (sCr, mg/dL), estimated glomerular filtration rate (eGFR, mL/min/1.73 m^2^) estimated using the CKD-EPI (Chronic Kidney Disease Epidemiology Collaboration) equation, creatinine clearance (ClCr, mL/min), albuminemia (Alb, g/dL), albumin creatinine ratio (ACR, mg/g), 24 h proteinuria (Pu24h, mg/24h), and urine alpha 1 microglobulin (α1m, mg/L).
3. B-mode renal ultrasound data: long- and short-axis imaging evaluation of both kidneys to determine the size, location, and echotexture. Length of the right and left kidneys (DL, cm) (maximum distance between poles in coronal section), wherein the normal ranges are 9–12 cm for the right kidney and 9–11 cm for the left kidney. The absence of masses, stones, calico-pyelectasis, and normal bladder emptying was verified.
4. Renal Doppler data: Through Doppler evaluation of the arcuate arteries at the level of the corticomedullary junction at the upper pole, in the central part, and the lower pole of the kidney, the right and left basal IR values were obtained (normal values range from 0.6–0.7).
5. Renal elastography data (Shear-Wave elastosonography GE Logiq E9): The probe was positioned perpendicular to the skin. When the image was stable, SWS measurement was initiated by fixing the region of interest (ROI) in the intermediate region of the right and left renal cortexes to ensure the shear waves were perpendicular to the main ultrasound beam, the vasa recta, and the loops of Henle. Five SWS measurements were taken, and the mean value was then used. Young’s modulus E (YM) was then obtained, registered, and measured in kPa. Following the elastography procedure, the patient was promptly referred for a biopsy on the same day.
6. Renal biopsy and histopathology: The procedure involved the extraction of two renal biopsy cores (about 1 cm in length and 2–3 mm in diameter). We followed the current guidelines for renal biopsy [9]. Small fragments were cut with a scalpel for electron microscopy and fluorescence. The remaining material was formalin-fixed and paraffin-embedded. Slides were cut and stained with hematoxylin–eosin, periodic acid Schiff, silver methenamine, and Masson’s trichrome. To assess the degree of chronicity, we used Sethi’s score, detailed in Table 1.
7. Outcome data: Data on the progression of renal pathology (sCr, p24h, eGFR) were collected at 1, 3, and 6 months. The group with AKD-P was further categorized into two subgroups—responders (R) or non-responders (NR)—based on their response to treatment, steroid therapy, and/or other immunosuppressive therapies as per the KDIGO guidelines for each nephropathy [10].

The HP underwent renal elastography conducted using the same procedure described in point 5 and in the same study period (May 2021–February 2023).

### Statistical Analysis of Data

The statistical analysis was carried out using the R programming language version 4.2.2 on a Mac OS operating system. The Shapiro test was employed to assess the normal distribution of numerical variables. This test is commonly used to determine whether or not a dataset is normally distributed. By using this information, we can make informed decisions about the statistical methods we use to analyze it. Variables that followed a normal distribution were described using means and standard deviations, while variables that did not follow a normal distribution were represented using median and interquartile range (IQR). To compare the YM and response to therapy, the Mann–Whitney test was employed. This test is a non-parametric alternative to the Student’s t-test and is used to compare two independent groups when the dependent variable is ordinal and non-normally distributed. The correlation between YM and laboratory data, ultrasound data, and biopsy data was expressed using the Spearman correlation coefficient. This coefficient is a non-parametric measure of correlation that is used to assess the strength and direction of a relationship between two variables.

A *p*-value of less than 0.05 was considered statistically significant, indicating that the results were unlikely to occur by chance. This means that there was a less than 5% chance that the observed differences were due to chance alone. To assess the factors associated with the YM of the biopsied side, stepwise multivariable logistic regressions were performed, adjusting for all the covariates. This allowed for a more accurate evaluation of the results, as it took into account multiple variables and their potential impact on the outcomes. In addition, to determine the differences between the YM of normal kidneys (HP) and AKD-P kidneys, the Mann–Whitney test was used after verifying the non-normal distribution via the Shapiro test.

## 3. Results

### Descriptive Analysis

The nephrological parameters at the time of the biopsy of the AKD-P were sCr 2.47 mg/dL (IQR 1.2–3.5) and eGFR 31 mL/min/1.73 m^2^ (IQR 13–61). The examined cohort of patients had mean histological scores, with a total histological score equal to 4.5 ± 2.9, a GS score equal to 1.2 ± 1.1, an IF score equal to 1.2 ± 0.85, a TA score equal to 1.2 ± 0.9, and a CV score equal to 0.78 ± 0.5.

Table 2 contains a detailed collection of the demographic, clinical, laboratory, ultrasound, and histological data for the AKD-P.

The group of 41 HP consisted of 21 women and 20 men, ranging in age from 21 to 78 years old, with an average age of 56 years. The patients’ BMI levels were within the normal range of 19 to 24. All patients included in this study exhibited normal renal function, with an estimated glomerular filtration rate (GFR) of between 90 and 120 mL/min/1.73 m^2^. This estimation was carried out by applying the CKD-EPI Creatinine Equation. We also evaluated the renal dimensions of the patients, finding that their kidneys’ bipolar diameter was between 9 to 12 cm for the right kidney and 9 to 11 cm for the left kidney, which is normal. Moreover, the cortical thickness in the middle third was measured and determined to be between 8.5 to 11 mm, which falls within the normal range.

Elastography data results are as follows:

The study examined two groups—AKD-P and HP—and measured the Young’s modulus (YM) values using elastography. The median YM value in the AKD-P group was 6.15 kPa (IQR 3.9), whereas in the HP group it was 12.2 kPa (IQR 2.7). The AKD-P group was further divided into two subgroups—R and NR—which consisted of 14 and 27 patients, respectively. The R subgroup had a median YM value of 7.4 kPa (IQR 3.7), whereas the NR subgroup had a median YM value of 5.6 kPa (IQR 2.8). The difference between the two subgroups was statistically significant (*p*-value = 0.037).

**Figure 1:**
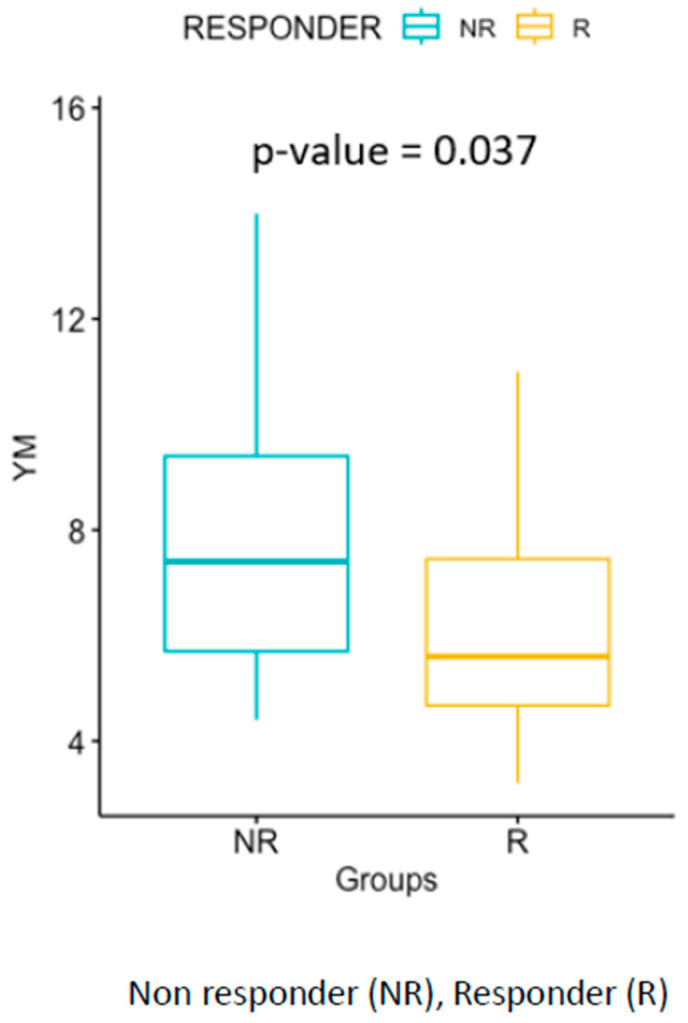
Significant difference between the elastography YM values of the R and NR groups.

The following analysis was carried out to achieve the study objectives through inferential methods.

(A) The correlation between renal elastography and Sethi score, SC, was investigated. The results showed no correlation between YM and the five chronic renal damage histological scores (ρ = −0.1254; *p* = 0.43444). The total chronic SC histological score correlated significantly with serum creatinine levels during the biopsy (*p* = 0.0007).
(B) The correlation between elastography and response to treatment was evaluated. It was found that patient responsiveness was associated with YM. The distribution of YM values showed lower values in the R subgroup and higher values in the NR subgroup. This distribution was statistically significant (*p*-value = 0.037). Figure 1 illustrates this correlation.
(C) The elastography data of the renal disease population were compared with those obtained from the healthy control group. The YM values identified in the HP were significantly higher than those in the AKD-P (*p*-value = 8.2 × 10^−12^).

## 4. Discussion

### 4.1. No Correlation Was Found between Renal Elastography and the Chronic Histological Score Determined through a Renal Biopsy

Our study showed that there was no correlation between histological chronic score and elastography stiffness in the AKD-P. This scenario has been proven in the liver, where acute damage can hinder the detection of fibrosis/cirrhosis using Fibroscan [11,12].

The inconsistent relationship between chronic histological changes and renal rigidity suggests that specific acute microenvironment alterations of the different compartments (glomerular, vascular, tubular, or interstitial) related to the underlying pathology, such as the extent of inflammation or microcirculation alterations, could contribute to renal stiffness [13,14].

Some researchers have suggested in preliminary studies that there might be a connection between stiffness and kidney injury caused by fibrosis [15,16].

The study conducted by Maralescu et al. [8] revealed that elastography lacks the ability to distinguish between various stages of fibrosis in patients diagnosed with glomerulonephritis. However, upon conducting a sub-analysis of the patients, the authors were able to differentiate those with mild to moderate fibrosis (<40%) from those with severe fibrosis (≥40%) using a cut-off of KPa < 20. It is noteworthy that the population of patients with severe fibrosis was limited to only 5 individuals, while those with mild to moderate fibrosis accounted for over 30 participants. Consequently, drawing a comparison between the two groups presents a challenge.

Nevertheless, in the realm of medical data, a disparity has been observed between the chronicity scores derived from tissue biopsy analysis and those obtained from elastography data. Newer biopsy studies have shown that the relationship between elasticity values and kidney damage is not straightforward [16,17]. Stiffness values may increase with mild to moderate fibrosis, but advanced fibrosis can lead to hypovascularization, resulting in a paradoxical decrease in stiffness. Research has found that in the later stages of chronic kidney disease, stiffness is more affected by poor blood flow than by how severe the fibrosis is [18]. According to the research conducted by Iyama et al., there appears to be no significant association between SWV and globally sclerotic glomeruli and interstitial fibrosis. This is particularly true when fibrotic conditions are of a low or intermediate grade [19].

It is of utmost importance to consider the occurrence of acute kidney damage in conjunction with the notable presence of fibrotic renal impairment while considering the pertinent context of hypovascularization.

The measurement of renal stiffness is considerably impacted by vascularity.

The kidneys are enclosed in a fibrous renal capsule, which reflects renal stiffness and is sensitive to changes in perfusion pressure. An increase in vascularization seems to be correlated with an increase in stiffness and, consequently, an increase in the propagation speed of the waves; on the contrary, a decrease in the blood supply to the kidney is responsible for a reduction in the stiffness of the renal parenchyma [20,21,22,23].

Based on the available evidence and our data, various factors, such as inflammation and fibrosis, can potentially impact the perfusion of the kidney [24].

It would be a mistake to oversimplify and assume that this issue is only confined to the fibrotic state.

### 4.2. Correlation between Elastography and Response to Treatment

Our analysis shows that lower YM values may be indicative of a more favorable therapeutic response. Though this information has yet to be published in the literature, it suggests that patients with lower YM values may experience a more severe acute inflammatory condition and may be more likely to respond positively to treatment. In cases where intrarenal edema is present, microcirculation can be restricted and blood flow to the affected area may be compromised, which can ultimately impact YM measurement [25]. It is imperative to conduct additional investigations to thoroughly explore the recently uncovered information, as it has not yet undergone comprehensive analysis within the existing corpus of research. It is crucial to acknowledge the significance of the information and unexpected preliminary data. Therefore, further investigation through extensive studies designed specifically for this purpose is necessary. This will allow for a comprehensive analysis of the results, facilitating the discovery of underlying factors contributing to the observed data.

### 4.3. Comparing the Elastography Data of AKD-P with the Data Obtained from HP

Compared to other organs like the liver, breasts, or thyroid, the stiffness values for the kidneys have not been universally agreed upon, making it challenging to determine a cut-off for distinguishing a diseased kidney from a healthy one. Our research shows that pathological kidneys tend to have lower YM values, which could be due to acute vascular impairment in the case of AKD-P or a fibrotic phenomenon in patients with CKD (although we did not evaluate this in our study, it has been well documented in other studies). This is because a healthy microvascular compartment contributes to stiffness. Similar conclusions were drawn by Bob and colleagues, who found that patients with interstitial fibrosis had significantly lower Kpa values compared to control groups [26]. Additionally, Hu et al. found lower SWV values in patients with immunoglobin A (IgA) compared to healthy volunteers [27].

## 5. Limitations of the Study

It is important to acknowledge that this study has certain limitations that need to be taken into consideration. The most significant limitations are the relatively small sample size and the inclusion of various pathological diagnoses, which could have had an impact on the statistical outcomes’ accuracy. The small sample size might have caused the results to be less representative of the population, as the sample might not have been diverse enough to represent the entire population. Additionally, the inclusion of different pathological diagnoses may lead to varying outcomes, impacting the accuracy of the results.

When interpreting the results obtained through elastography, it is important to consider the intrinsic limitations of this technique. These limitations include renal anisotropy, variations in organ depth, changes in blood flow, and the operator-dependent nature of the technique. The last item relates to the fact that this technique has yet to be standardized, which may result in inconsistent results among different operators. This technique is highly dependent on the skills and experience of the operator, making it essential to ensure that the operator is qualified and experienced in performing the procedure. Additionally, it is advisable to consider the aforementioned limitations when interpreting the results.

## 6. Conclusions

A kidney biopsy is highly valuable for both the diagnosis and examination of fibrotic aspects, and it is a challenging procedure to replace. According to our data, while elastography may not be as exhaustive or accurate as a kidney biopsy, it presents an opportunity to gain a preliminary understanding of a treatment response. Renal stiffness is subject to various factors, including fibrotic and inflammatory mechanisms. Understanding the connection between these mechanisms and the elastography pathway can provide valuable insights. Therefore, it is crucial to carefully examine elastography data in acute kidney injury conditions and compare them with those relating to healthy patients to determine normal reference values. Our research marks the first attempt to assess the predictive value of renal YM in terms of response to nephrological treatment. As the present study represents the first identification of this particular aspect, it would be prudent to corroborate these findings with further in-depth and targeted investigations.

## Figures and Tables

**Table 1:** Sethi score (2017) [2].

Tissue Compartment	Score			
	0	1	2	3
Glomerulosclerosis (GS)	<10%	10–25%	26–50%	>50%
Interstitial fibrosis (IF)	<10%	10–25%	26–50%	>50%
Tubular atrophy (TA)	<10%	10–25%	26–50%	>50%
Arteriosclerosis (CV)	Intimal thickness < thickness of media	Intimal thickness ≥ thickness of media		

The total chronicity score is the sum of the chronicity scores of each compartment (0–10).

**Table 2:** Clinical, laboratory, ultrasound, and histological AKD-P data.

Male, n (%)	29 (72)
Female, n (%)	12 (29)
Age (years), mean ± SD	41 ± 16
BMI (kg/m^2^), mean ± SD	27 ± 3
Renal disease	
TIN, n (%)	10 (24.4)
IgA, n (%)	5 (12.2)
DN, n (%)	4 (9.7)
ANCA, n (%)	4 (9.7)
NFA, n (%)	4 (9.7)
LES, n (%)	3 (7.3)
MCD, n (%)	3 (7.3)
FSGS, n (%)	2 (4.8)
GNM, n (%)	2 (4.8)
RA, n (%)	2 (4.8)
ICN, n (%)	1 (2.4)
IGMM, n (%)	1 (2.4)
Laboratory data	
sCr (mg/dL), median (IQR)	2.47 (1.2–3.5)
eGFR (mL/min/1.73 m^2^), median (IQR)	31 (13–61)
ClCr (mL/min), median (IQR)	33 (18–59)
Alb(g/dL), mean ± SD	3 ± 0.5
ACR (mg/g), median (IQR)	1505 (0–6000)
Pu24h (mg/24 h), median (IQR)	2.6 (0–6500)
α1m (mg/L), median (IQR)	48 (5–130)
Renal ultrasound data	
DL sin (cm) mean ± SD	11 ± 0.8
DL dx (cm) mean ± SD	11 ± 1
RI sin, (cm) mean ± SD	0.62 ± 0.05
RI dx, (cm), mean ± SD	0.62 ± 0.04
Histological scores	
Total histological score, mean ± SD	4.5 ± 2.9
GS, mean ± SD	1.2 ± 1.1
IF, mean ± SD	1.2 ± 0.85
TA, mean ± SD	1.2 ± 0.9
CV, mean ± SD	0.78 ± 0.5

Body mass index (BMI), tubulointerstitial nephritis (TIN), IgA nephropathy (IgA), diabetic nephropathy (DN), antineutrophilic cytoplasmic antibody-associated vasculitides (ANCA), acute tubular necrosis (NFA), lupus nephritis (LES), minimal-change disease (MCD), focal segmental glomerulosclerosis (FSGS), membranous nephropathy (GNM), renal amyloidosis (RA), Immune Complex Nephritis (ICN), IgM nephropathy (IGMN), serum creatinine (sCr), estimated glomerular rate (eGFR), Creatinine Clearance (ClCr), serum albumin (Alb), albumin-to-creatinine ratio (ACR), proteinuria of 24H (Pu24H), alpha 1 microglobulin urine (α1m), longitudinal diameter (DL), renal intraparenchymal resistive index (RI), Glomeulosclerosis (GS), interstitial fibrosis (IF), tubular atrophy (TA), and arteriosclerosis (CV).

## Data Availability

The data underlying this article will be shared on reasonable request made to the corresponding author.
